# Non-invasive predictors of ICU admission and mortality in initially asymptomatic COVID-19 patients

**DOI:** 10.1186/s43168-022-00156-4

**Published:** 2022-10-27

**Authors:** Riham Hazem Raafat, Mohamed Alboraie, Sally Waheed Elkhadry, Mostafa Abdelnasier, Mohamed Ahmed Hashish, Yahya Ahmed Almansoury, Noha Yousef, Omar Elshaarawy, Ahmad Madkour

**Affiliations:** 1 Chest Department, Ain Shams University, Cairo, Egypt; 2 Department of Internal Medicine, Al-Azhar University, Cairo, Egypt; 3 Epidemiology and Preventive Medicine Department, National Liver Institute Menoufia University, Menoufia, Egypt; 4 Internal Medicine Department, Ain Shams University, Cairo, Egypt; 5 Internal Medicine Department, Gastroenterology and Hepatology Division, Qena University Hospital, South Valley University, Qena, Egypt; 6 Hepatology, Gastroenterology and Liver Transplantation Department, National Liver Institute, Menoufia University, Menoufia, Egypt; 7 Department of Gastroenterology, Royal Liverpool University Hospital, NHS, Liverpool, UK; 8 Endemic Medicine Department, Faculty of Medicine, Helwan University, Cairo, Egypt

**Keywords:** SARS-CoV-2, COVID-19, Intensive care units, Asymptomatic Infections, Mortality

## Abstract

**Background:**

Coronavirus disease 2019 (COVID-19) can present with pulmonary and non-pulmonary manifestations, or it may be asymptomatic. Asymptomatic patients have a major impact on transmission of the disease, and prediction of their outcome and prognosis is challenging. We aim to identify the predictors of intensive care unit (ICU) admission and mortality in hospitalized COVID-19 patients with initially asymptomatic presentation.

**Methods:**

This was a prospective multicenter study using cohort data that included all admitted patients aged 21 years and above, with different clinical presentations other (than pulmonary manifestation) and were discovered to have COVID-19. Demographic data, clinical data and progression were reported. Univariate analysis and logistic regression analysis were performed to predict ICU admission and mortality during hospitalization.

**Results:**

One hundred forty-nine consecutive patients, 92 (61.7% males) were included in our study, Median age (IQR) was 59.00 (43–69]. Only 1 patient (0.7%) had a contact with a confirmed case of COVID-19. 58 patients (39%) were admitted to ICU and 22 patients (14.8%) have died. High ferritin level (more than 422.5), low oxygen saturation (less than 93%), and in need of non-invasive ventilation (NIV) have 3.148, 8.159 and 26.456 times likelihood to be admitted to ICU, respectively. Patients with high CO-RADS, low oxygen saturation (less than 92.5%), and in need for mechanical ventilation (MV) have 82.8, 15.9, and 240.77 times likelihood to die, respectively.

**Conclusion:**

Initially asymptomatic hospitalized patients with COVID-19 have a great impact on health system with high ICU admission and mortality rate. We identified the predictors that may help in early management and improving prognosis.

**Trial registration:**

Trial was registered in Clinicaltrials.gov, registration number is NCT05298852, 26 March 2022, retrospectively registered.

## Introduction

Coronavirus disease 2019 (COVID-19) was first discovered at Wuhan, China in December 2019, declared by World Health Organization (WHO) as an outbreak on 20th January, 2020 and as a pandemic in 11th March, 2020. Up till now, there are more than 130 million confirmed cases and 2.84 million deaths, making it the most aggressive pandemic worldwide [1]. This terrifying spread has exhausted health systems and medical resources [2]. A lot of efforts are made to reinforce medical resources, alleviate health systems stress and control COVID-19 pandemic [3]. However, it is challenging and difficult to achieve control of the pandemic, especially with limited resources situations.

Coronavirus has a wide variety of presentations, most of cases have mild to moderate symptoms, while 14% have severe symptoms [4, 5]. At least one third of infected patients are asymptomatic [6–8], whereas those with asymptomatic infection cause spread of infection [8–10]. Another category of infected patients develop symptoms later are called “pre-symptomatic” and can also spread the virus [10]. Large category of patients has non-pulmonary symptoms, 11.8% develop cardiac damage with elevated cardiac troponin I or cardiac arrest with no previous history of cardiovascular disease [11]. 3% of admitted patients with COVID-19 have digestive symptoms without respiratory symptoms [12], while another study discovered 7% of patients with digestive symptoms [13]. Many acute kidney injury (AKI) cases are reported in patients with COVID-19 [13–16].

High-resolution CT chest (HRCT) proved its significance in diagnosing COVID-19 [17]. Findings in most patients are bilateral multilobar ground-glass opacities with a peripheral, asymmetric, and posterior distribution [18]. Comparison between chest CT results to PCR revealed that imaging is less specific for the infection, but it is faster and more sensitive [19].

There is variation in the incidence of asymptomatic COVID-19 due to different screening policies among countries [8]. A wide range of incidents from 18 to 81% is reported [20–22]. This difference in incidence may be due to limited resources in some countries for screening. Although the Centre for Disease Control and Prevention recommended limited testing for asymptomatic patients; it now recommends widespread testing, regardless of signs or symptoms to mitigate the transmission of SARS-CoV-2 [23]. Asymptomatic patients have the same infectivity as symptomatic ones [24]. Also, it is reported that asymptomatic patients have viral load similar to symptomatic patients [25]. Therefore, asymptomatic COVID-19 patients carry a great risk in the transmission of infection and burden. In this study, we aim to identify the predictors of intensive care unit (ICU) admission and mortality in hospitalized COVID-19 patients with initially asymptomatic presentation.

## Methods

### Study population

This is a prospective multicenter study that included patients aged more than 21 years old with different clinical presentations, other than pulmonary manifestation, admitted to different healthcare facilities from March to December 2020. HRCT scan of the chest in order to detect COVID-19 patients was offered after signing an informed consent. Demographic data, clinical presentations, laboratory data, oxygen saturation, radiological findings in HRCT scan of the chest, SARS-CoV-2 PCR results and the need for mechanical ventilation were reported. Effects of different baseline characteristics, findings in HRCT scan of the chest on patient outcomes were analyzed.

### Data collection

Data were collected from participating institutions (Ain shams University, Al-Azhar University, National Liver Institute Menoufia University, and Helwan University). Clinical presentations including history of potential source of infection, contact with COVID-19 patients, clinical examination findings were registered. HRCT scan of the chest for all patients upon admission and scoring system for severity of lung affection was performed. Patients with positive HRCT chest findings were investigated with PCR result for COVID-19. Complete blood picture with differential and serum ferritin level levels were examined. Baseline oxygen saturation on room air (RA), follow-up of oxygen status, need for oxygen, non-invasive ventilation (NIV), mechanical ventilation (MV), and need for ICU admission were reported.

### Outcomes

The primary outcome was intensive care unit admission, use of non-invasive ventilation or mechanical ventilation. The secondary outcome was mortality.

### Sample size

Using Epi Info program for sample size calculation and assuming the prevalence of asymptomatic COVID-19 patients was ranging from 10% (*p* = 0.1), with a margin of error 5% precision 5% (precision *d* = 0.05), at 95% confidence level, *Z* statistic for a 95% level of confidence (*Z* = 1.96) and the used equation (*n* = {Z2 × *P* × (1 − *P*)}/d2), a sample size of at least 139 patients were needed. All patients fulfilling the inclusion criteria were included in the study till completion of the sample size.

### Statistical analysis

Data were collected and analyzed using SPSS (Statistical Package for Social Science) program for statistical analysis, (version 23; Inc., Chicago, IL). Quantitative data were presented as mean, SD, and range. Qualitative data were presented as frequency and percent. Chi-square test was used to measure association between qualitative variables. Fisher exact test was used for 2 × 2 qualitative variables when more than 25% of the cells have expected count less than 5. Mann–Whitney test was used to compare mean and SD of 2 sets of quantitative data when these data were not normally distributed. Logistic regression model was used to give adjusted odds ratio and 95% confidence interval of the effect of the different risk factors for subjects in the study. The receiver operating characteristic (ROC) curve was done to detect the cut-off value with the highest sensitivity and specificity. Sensitivity, specificity, positive and negative predictive values, and diagnostic accuracy were calculated. *P* value was considered statistically significant when it was less than 0.05.

### Ethical consideration

The project ethical approval was obtained from Faculty of Medicine, Al-Menoufia University Ethics Committee. Confidentiality of data was ensured throughout the study. Informed consent was obtained from all participants following the provision of an explanation of the study rationale and procedures.

## Results

Our study included 149 consecutive patients admitted to hospital for non-pulmonary reasons during the COVID-19 pandemic. Median age (IQR) was 59.00 (43–69) and 92 (61.7%) of patients were males. Only 1 patient (0.7%) had a contact with a confirmed case of COVID-19 and 38 patients (25.5%) were smokers. Symptoms were mainly non-pulmonary in 117 patients (78.5%), and 133 patients (89.3%) had fever. Clinical presentations and indications for admission of the studied group are presented in (Tables 1 and 2). Upon admission, median oxygen saturation (IQR) on room air was 95(92–97). Initial laboratory investigations showed that 139 patients (93.3%) had lymphopenia, and median baseline serum ferritin level was 455.00 (IQR 205–910). Baseline high-resolution computed tomography of the chest (HRCT) using The Coronavirus disease 2019 (COVID-19) Reporting and Data System (CO-RADS) revealed that 91 patients (61.1%) had CO-RADS grade 5 (CO-RADS 5), while 55 patients (36.9%) had CO-RADS 4 and 3 patients (2.0%) had CO-RADS 3. Initially, 88 patients (59.1%) were admitted to regular in-patient ward. During hospital course, 58 patients (38.9%) were admitted to the intensive care unit, 20 patients (13.4%) needed non-invasive ventilation, 26 (17.4%) needed mechanical ventilation, and 22 patients (14.8%) died.

**Table 1:** Diagnostic test accuracy for prediction of admission to intensive care unit

	**Best cutoff**	**Sensitivity**	**Specificity**	**Positive predictive value**	**Negative predictive value**	**Accuracy**	**AUC**	***P* value**
So2 on RA (93%)	93	70.690% (57.27% to 81.91%)	84.615% (75.536% to 91.326%)	74.545% (63.76% to 82.98%)	81.915% (75.053% to 87.211%)	79.195% (71.787% to 85.405%)	0.874	0.0001
Ferritin	422.5	70.69% (57.27% to 81.91%)	58.24% (47.43% to 68.50%)	51.90% (44.58% to 59.14%)	75.71% (66.84% to 82.82%)	63.09% (54.80% to 70.84%)	0.696	0.0001
NIV	–	31.03% (19.54% to 44.54%)	97.80% (92.29% to 99.73%)	90.00% (68.44% to 97.39%)	68.99% (65.12% to 72.61%)	71.81% (63.87% to 78.87%)	–	–
The 3 factors in series	–	19.403% (10.756% to 30.891%)	100.00% (96.03% to 100.00%)	100.00%	62.76% (59.97% to 65.46%)	65.823%% (57.866% to 73.173%)	–	–

**Table 2:** Multivariate logistic regression model for prediction of admission to ICU

Multivariate logistic regression model	Sig	OR 95% CI
Age	**0.333**	**1.014** **(0.986–1.042)**
Sex	**0.654**	**0.790** **(0.283–2.208)**
CORAD	**0.782**	**0.648** **(0.03–13.917)**
Lymph	**0.056**	**0.113** **(0.012–1.059)**
ferritin (high level)	**0.016**	**3.148** **(1.241–7.985)**
Presence Resp Symp	**0.527**	**1.449** **(0.459–4.576)**
Smoker	**0.456**	**1.516** **(0.508–4.529)**
So2R (low saturation93)	**0.000**	**8.159** **(3.034–21.941)**
Fever	**0.140**	**0.218** **(0.029–1.644)**
NIV(1)	**0.003**	**26.456** **(2.945–237.636)**
Constant	**0.162**	**0.087**

^a^Variable(s) entered on step 1: age, sex, CORAD, lymph, ferritin G, smoker, presence Resp S, So2R (low saturation), Fever, NIV

**Fig. 1:**
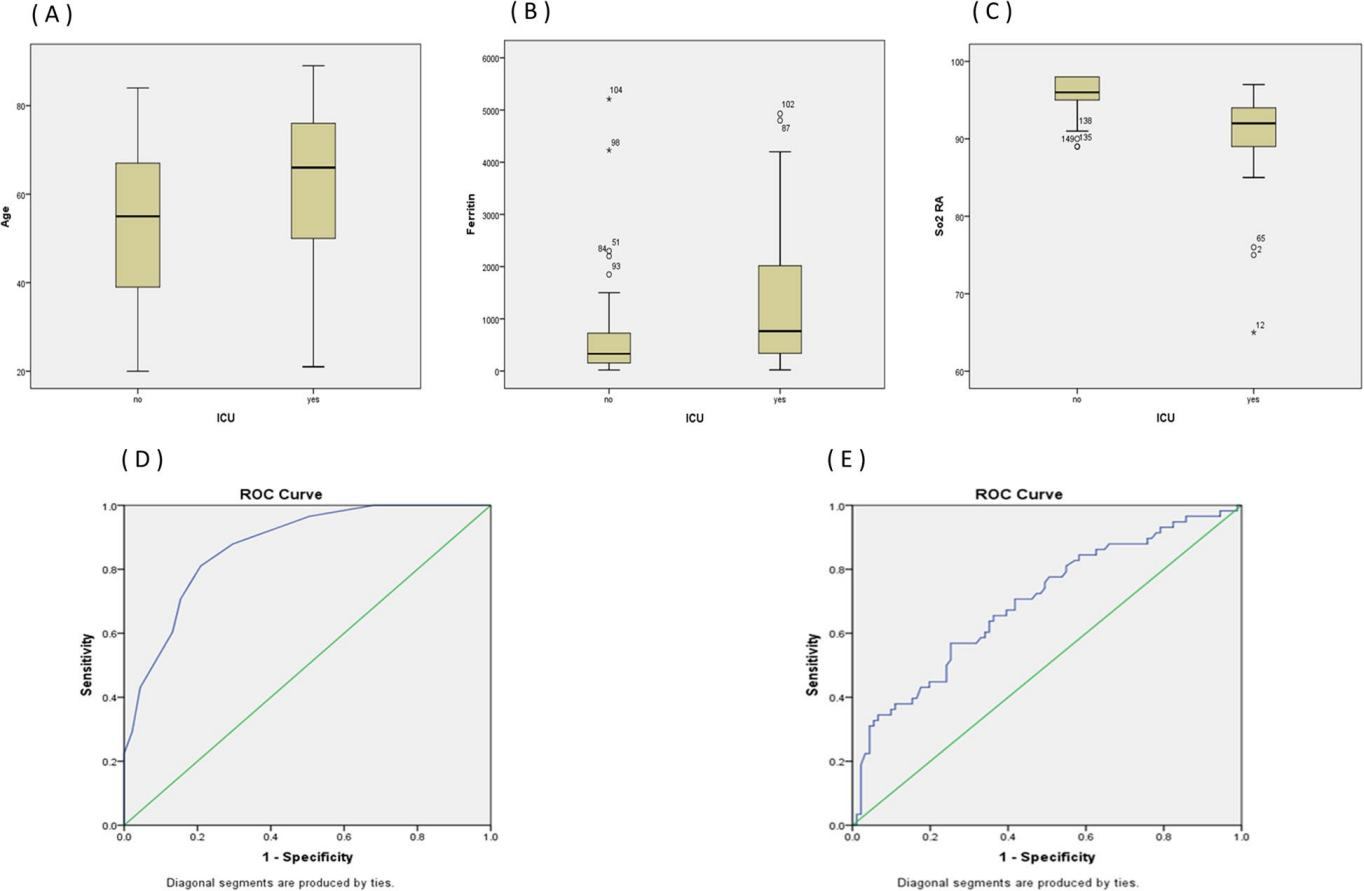
**A **Boxplot for age distribution in relation to ICU admission. **B **Boxplot for serum ferritin distribution in relation to ICU admission. **C **Boxplot for baseline oxygen saturation distribution in relation to ICU admission. **D **Receiver operator characteristics (ROC) curve for baseline oxygen saturation in patients admitted to ICU. **E **Receiver operator characteristics curve for Serum Ferritin in patients admitted to ICU

Univariate analysis showed that older patients (mean age of 61.66 years old or older), smokers, patients with higher serum ferritin (mean value of 1305.09 or higher) and lower oxygen saturation (mean baseline oxygen saturation of 90.72 or less), and those in need for non-invasive ventilation were predictors of admission to intensive care unit (Table 3). The best cut-off level for baseline oxygen saturation to predict admission to intensive care unit was 93%. The best cut-off values for serum ferritin to predict admission to intensive care unit was 422.5. Table 1 shows the diagnostic accuracy for baseline oxygen saturation, serum ferritin, and non-invasive ventilation for admission to intensive care unit. Combining these three factors was highly specific (100%) with a positive predictive value of 100% for prediction of admission to intensive care unit. Figure 1 shows boxplots for age, serum ferritin and oxygen saturation on room air in relation to ICU admission. The area under receiver operator characteristics (ROC) curve for baseline oxygen saturation in patients admitted to ICU was 0.874 and that under ROC curve for serum ferritin was 0.696 (Fig. 1).

**Table 3:** Diagnostic test accuracy for prediction of mortality

	**Best cutoff**	**Sensitivity**	**Specificity**	**Positive predictive value**	**Negative predictive value**	**Accuracy**	**AUC**	***P* value**
**So2 RA**	92.5	90.909% (70.839% to 98.879%)	78.740% (70.596% to 85.498%)	42.553% (34.075% to 51.493%)	98.039% (93.005% to 99.471%)	80.537% (73.259% to 86.561%)	0.92	0.0001
**Ferritin**	1020	59.09% (36.4% to 79.3%)	85.04% (77.6% to 90.7%)	40.625% (28.483% to 54.032%)	92.308% (87.841% to 95.223%)	81.208% (73.999% to 87.135%)	0.726	0.0013
**CO-RAD** Highly suspicious (4,5)	–	95.455% (77.156% to 99.885%)	98.425% (94.427% to 99.809%)	91.304% (72.579% to 97.655%)	99.206% (94.850% to 99.882%)	97.987% (94.229% to 99.583%)	–	–
**MV**	–	90.909% (70.839% to 98.879%)	95.276% (90.001% to 98.247%)	76.923% (60.153% to 88.039%)	98.374% (94.162% to 99.561%)	94.631% (89.695% to 97.654%)	–	–
**The 3 factor in series** **So2 RA, MV, CORAD**	–	81.818% (59.715% to 94.813%)	98.425% (94.427% to 99.809%)	90.000% (69.173% to 97.304%)	96.899% (92.793% to 98.699%)	95.973% (91.442% to 98.508%)	–	–

Logistic regression was performed to ascertain the effects of different factors on the likelihood of ICU admission. Patients with high ferritin level (more than 422.5) were 3.148 times more likely to be admitted to ICU than patients with lower levels. Patients with low oxygen saturation (less than 93%) were 8.159 times more likely to be admitted to ICU than patients with higher saturation. Patients needed NIV were 26.456 times more likely to be admitted to ICU (Table 2).

Univariate analysis showed that older age, history of smoking, high serum ferreting level, presence of fever, respiratory symptoms, low oxygen saturation and the need for non-invasive ventilation, mechanical ventilation or admission to intensive care unit were significant predictors for mortality (Table 4).

**Table 4:** Multivariate logistic regression model for prediction of mortality

Multivariate logistic regression model for non survivors	Sig	OR 95% CI
Age	**0.983**	**0.999** **(0.933**–**1.071)**
Sex	**0.429**	**0.363** **(0.029**–**4.478)**
Lymph	**0.927**	**0.793** **(0.006**–**113.114)**
CO-RADS Highly suspicious(4,5)	**0.039**	**82.811** **(1.248**–**5495.935)**
Ferritin	**0.563**	**1.968** **(0.199**–**19.488)**
Presence RespS	**0.636**	**0.556** **(0.049**–**6.333)**
Smoker	**0.206**	**8.400** **(0.311**–**226.580)**
So2RA (low saturation 92.5)	**0.039**	**15.872** **(1.154**–**218.249)**
Fever	**0.358**	**47.185** **(0.013**–**175,137.406)**
ICU	**0.996**	**28,928,705.328** **(0.000**
MV	**0.003**	**240.769** **(6.777**–**8554.201)**
Constant	**0.995**	**0.000**

^a^Variable(s) entered on step 1: age, sex, lymph, CORAD, ferritin, presence respiratory signs and symptoms, smoker, So2RA92.5, fever, ICU, MV

**Fig. 2:**
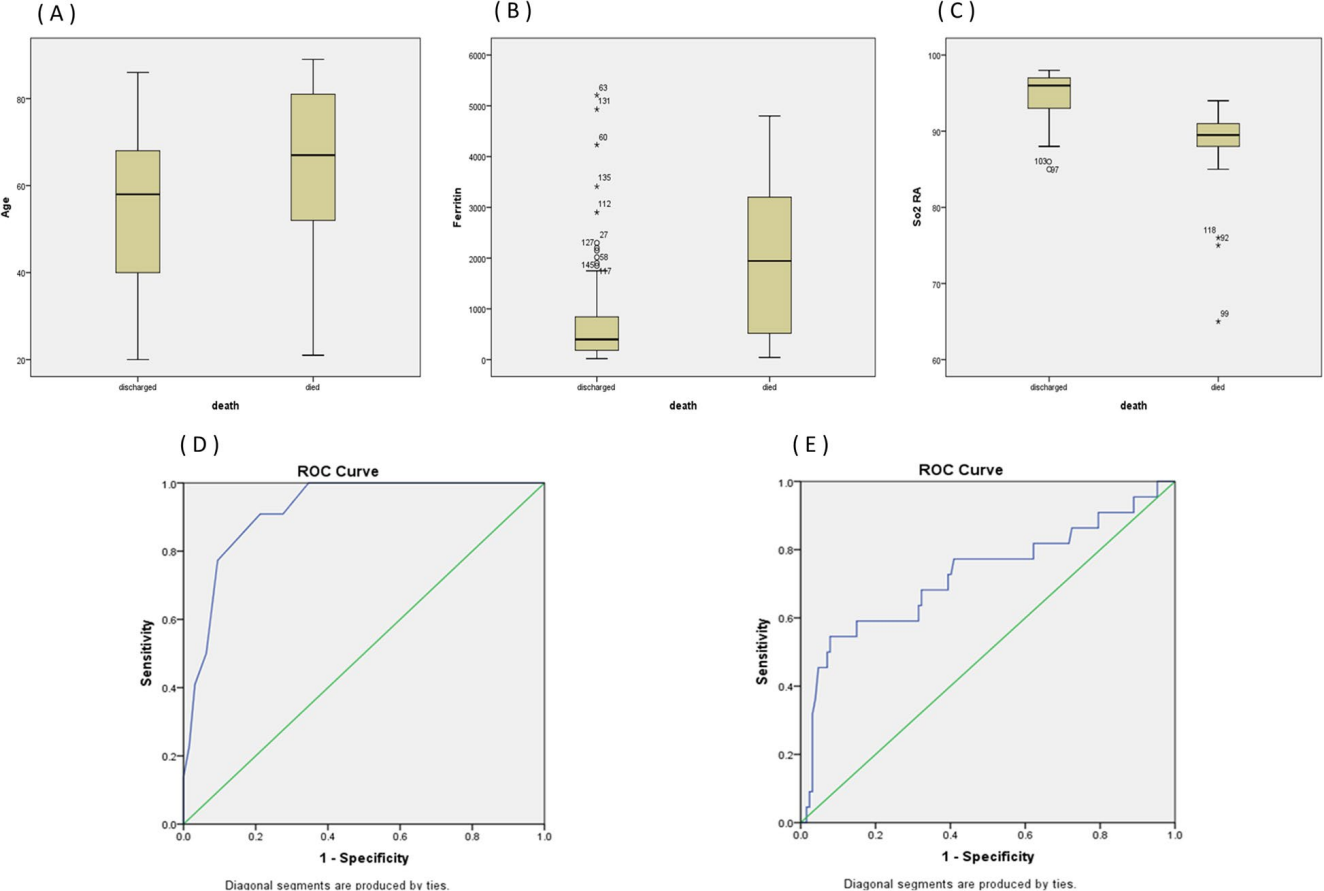
**A** Boxplot for Age Distribution in relation to mortality. **B** Boxplot for serum ferritin distribution in relation to mortality. **C** Boxplot for baseline oxygen saturation distribution in relation to mortality. **D** Receiver operator characteristics (ROC) curve for baseline oxygen saturation in non-surviving patients. **E **Receiver operator characteristics curve for serum ferritin in in non-surviving patients

The best cut-off level for baseline oxygen saturation to predict mortality was 92.5%. The best cut-off values for serum ferritin to predict mortality were 1020. Table 3 shows the diagnostic accuracy for baseline oxygen saturation, serum ferritin, CO-RADS grade (highly suggestive of COVID-19) and mechanical ventilation for prediction of mortality. Combining oxygen saturation, CO-RADS grade (highly suggestive of COVID-19) and mechanical ventilation was highly specific (98.4%) with a positive predictive value of 90% for prediction of mortality (Table 3). Figure 2 shows boxplots for age, serum ferritin and oxygen saturation on room air in relation to mortality. The areas under receiver operator characteristics (ROC) curve for baseline oxygen saturation in non-surviving patients was 0.874 and that under ROC curve for serum ferritin was 0.696 (Fig. 2).

Logistic regression was performed to ascertain the effects of different factors on the likelihood mortality. Patients with high suspicious CO-RADS grade were 82.8 times more likely to die than patients with intermediate suspicious CO-RADS grade. Cases with low oxygen saturation (less than 92.5%) were 15.9 times more likely to die than patients with higher saturations. Patients who required mechanical ventilation were 240.77 times more likely to die than patients who did not require mechanical ventilation (Table 4).

## Discussion

This study examined the clinical outcomes of initially asymptomatic COVID-19 patients who were admitted to the hospital with medical or surgical problems and were diagnosed to have COVID-19 after routine testing with HRCT chest scan and nasal swab for severe acute respiratory syndrome coronavirus 2 (SARS-CoV-2) by reverse transcriptase-polymerase chain reaction (RT-PCR). We included 149 patients who initially had non-pulmonary symptoms. The study population was admitted to the hospitals with different clinical presentations, like surgical indications, diabetic complications, electrolytes disturbances, blood transfusion, and cardiac complications.

Little is known about asymptomatic and initially asymptomatic COVID-19 patients; most of published data discuss the risk and mortality of symptomatic COVID-19 patients. In our study, we examined the risks and non-invasive predictors for ICU admission and mortality in initially asymptomatic patients who were admitted to different departments with variable presentations other than pulmonary symptoms. During hospital stay, 40% of patients were admitted to ICU. Age, smoking, ferritin level, development of respiratory symptoms, oxygen saturation on room air, and the need for NIV were the main predictors for the ICU admission. On the other hand, male sex, lymphocytopenia, CT findings, and fever had no significance effect with respect to ICU admission. Testing the accuracy of serum ferritin level, oxygen saturation, and the need for NIV in series revealed very high specificity and positive predictive value with low sensitivity. A logistic regression analysis to ascertain the predictors for ICU admission showed that cases with high ferritin level (more than 422.5) have more than three times vulnerability to be admitted to ICU, while cases with low SO2 (≤ 93%) have more than eight times likelihood to be admitted to ICU and cases needed NIV on hospital admission have more than 26 times possibility to be admitted to ICU.

A large study has examined the clinical outcomes of initially asymptomatic patients with COVID-19 and compared the outcomes to symptomatic COVID-19 patients. They calculated an age-adjusted Charlson comorbidity index score (CCIS) from a weighted index consisted of age and the number and seriousness of comorbid diseases which revealed that the age is a highly significant predictor in both ICU admission rate and mortality. On the contrary, they revealed the significance of male sex, anaemia, and lymphocytopenia in the prediction of ICU admission [26]. These different results may be because they have studied a larger population who developed mainly pulmonary symptoms, while in our study, we studied patients with non-pulmonary symptoms. Petrilli et al. also found that age is a strong predictor for hospital admission and critical illness in COVID 19 patients [27]. Regarding high ferritin level, a study included 141 patients with COVID‐19 reported that severe cases and ICU patients have higher ferritin levels than mild cases (2.6 times and 5.8 times, respectively) [28]. More than third of the patients who are admitted ICU developed respiratory symptoms, unlike the non-ICU patients, and this is consistent with the findings of Arentz et al. [29]. Oxygen saturation (SO_2_) on room air (RA) at admission revealed its significance in predicting ICU admission among patients, with *P* value < 0.0001. A study performed to examine the early predictive factors for progression from severe type to critically ill type infection with COVID-19 revealed that patients admitted with baseline SO_2_ ≤ 93% have tendency to progress to critical care unit [30]. In our study, fever was not a predictor for ICU admission on the contrary to the findings in Ioannou et al. who demonstrated that patients with fever had a higher risk of development of critical illness and mechanical ventilation than those without fever [31]. Also, we demonstrated that sex was not a predictor for ICU admission. In contrast, Sokolowska et al. demonstrated increased male vulnerability to infection than females [32]. Another comprehensive study showed that male sex is a risk factor for ICU admission [26]. Contrary to our study, Yang et al. reported that most of the critically ill adult patients had lymphocytopenia with no significant difference between survivors and non survivors [14]. This difference may be because they have studied critically ill patients who present initially with pulmonary symptoms, a group excluded from our study. A study by Zhang et al. reported that CT findings (CO-RAD) were higher in severe patients than mild to moderate patients [33], which is not compatible with the findings of our study, as there was no significant difference between those admitted to ICU and patients who did not need ICU admission with respect to CT findings. This difference may be attributed to the timing of doing CT chest in our study. It was at admission when the patients were asymptomatic and most of them had no follow-up, so the progression to more severe CT findings could not be recorded. Most of our patients who needed NIV progressed to critical illness and needed ICU admission. This corresponds to the findings of a study that examined the use of NIV in COVID-19 patients and concluded that the use of NIV without achieving improvement delayed the decision for mechanical ventilation, causing progression to respiratory failure and even death [34]. Another opinion declared that the early use of NIV reduces deterioration and the need for MV [35]. Age, smoking status, development of respiratory symptoms, fever, oxygen saturation on room air ≤ 92.5%, need for NIV, ICU admission, and mechanical ventilation (MV) use were predictors for mortality in our study. Need for MV, serum ferritin level ≥ 1020, and SO_2_ ≤ 92.5% in a series of COVID-19 patients showed very high sensitivity, specificity, positive predictive value, and negative predictive value with accuracy of 95.973% for prediction of mortality.

A logistic regression analysis to ascertain the predictors of mortality in asymptomatic patients with COVID-19 showed that cases with highly suspicious CT findings (CO-RAD 5) have more than eighty times risk for death than intermediately suspicious CORADS, cases with low SO_2_ saturation (≤ 92.5%) have more than fifteen times risk to die than patients with higher saturations, and MV Cases have 240.769 times risk to die compared to patients did not need MV. An Italian study tested early warning signs in COVID-19 patients through different scores like NEWS, NEWS2, NEWS-C, MEWS, qSOFA, and REMS for predicting ICU admission and death at 48 h and 7 days. They concluded that the national early warning score (NEWS), which is a score using vital data like conscious level, temperature, heart rate, respiratory rate, blood pressure, SO_2_, and any supplemental oxygen need, was the most accurate predictor for ICU admission, while rapid emergency medicine score (REMS), another score using age, heart rate, blood pressure, SO_2_, respiratory rate, and conscious level, was the most accurate predictor for death [36]. Another study revealed that patients with older age (> 65 years), comorbidities, developed ARDS and needed MV were at increased risk of death [14]. Another opinion support these results concluded that the most accurate predictor for death is age and comorbidities [26, 27, 31].

We concluded that initially asymptomatic hospitalized patients with COVID-19 have a great impact on health system with high ICU admission and mortality rate. We identified the predictors that may help in early management and improving prognosis.

## Limitations

This study has some limitations; for instance, the relatively small number of patients may limit its generalizability. Missing data such as follow-up of CT images to identify changes and progression, treatment data that may impact outcome, and lack of pediatric patients can also be added to the limitations of this study.

## Data Availability

Not applicable.

## Abbreviations

COVID-19: Coronavirus disease 2019
ICU: Intensive care unit
NIV: Non-invasive ventilation
MV: Mechanical ventilation
AKI: Acute kidney injury
HRCT: High-resolution CT chest
ROC: The receiver operating characteristic
CO-RADS: (COVID-19) Reporting and Data System
CCIS: Charlson comorbidity index score
SO_2_: Oxygen saturation
RA: Room air

## References

[1] (JHU) C-19 D by the C for SS and E (CSSE) at JHU. “COVID-19 Dashboard by the Center for Systems Science and Engineering (CSSE) at Johns Hopkins University (JHU)”. ArcGIS. Johns Hopkins University. Available from: https://gisanddata.maps.arcgis.com. [cited 2021 Apr 3]

[2] Wang C, Horby PW, Hayden FG, Gao GF (2020). A novel coronavirus outbreak of global health concern. Lancet.

[3] Ranney ML, Griffeth V, Jha AK (2020). Critical supply shortages — the need for ventilators and personal protective equipment during the Covid-19 pandemic. N Engl J Med.

[4] Grant MC, Geoghegan L, Arbyn M, Mohammed Z, McGuinness L, Clarke EL, et al. The prevalence of symptoms in 24,410 adults infected by the novel coronavirus (SARS-CoV-2; COVID-19): a systematic review and meta-analysis of 148 studies from 9 countries. PLoS One. 2020;15(6 June). 10.1371/journal.pone.0234765 10.1371/journal.pone.0234765 PMC7310678 32574165

[5] Pascarella G, Strumia A, Piliego C, Bruno F, Del Buono R, Costa F (2020). COVID-19 diagnosis and management: a comprehensive review. J Intern Med.

[6] Oran DP, Topol EJ (2021). The proportion of SARS-CoV-2 infections that are asymptomatic: a systematic review. Ann Intern Med.

[7] Liu Y, Yan LM, Wan L (2020). Viral dynamics in mild and severe cases of COVID-19. Lancet Infect Dis.

[8] Gao Z, Xu Y, Sun C, Wang X, Guo Y, Qiu S, Ma K (2021). A systematic review of asymptomatic infections with COVID-19. J Microbiol Immunol Infect.

[9] Oran DP, Topol EJ (2020). Prevalence of Asymptomatic SARS-CoV-2 Infection : A Narrative Review. Ann Intern Med.

[10] Furukawa NW, Furukawa NW, Brooks JT, Sobel J (2020). Evidence supporting transmission of severe acute respiratory syndrome coronavirus 2 while presymptomatic or asymptomatic. Emerg Infect Dis.

[11] Clerkin KJ, Fried JA, Raikhelkar J, Sayer G, Griffin JM, Masoumi A (2020). COVID-19 and cardiovascular disease. Circulation.

[12] Pan L, Mu M, Yang P, Sun Y, Wang R, Yan J (2020). Clinical characteristics of COVID-19 patients with digestive symptoms in Hubei, China: a descriptive, cross-sectional, multicenter study. Am J Gastroenterol.

[13] Huang C, Wang Y, Li X, Ren L, Zhao J, Hu Y (2020). Clinical features of patients infected with 2019 novel coronavirus in Wuhan, China. Lancet.

[14] Yang X, Yu Y, Xu J, Shu H, Xia J, Liu H (2020). Clinical course and outcomes of critically ill patients with SARS-CoV-2 pneumonia in Wuhan, China: a single-centered, retrospective, observational study. Lancet Respir Med..

[15] Wang D, Hu B, Hu C, Zhu F, Liu X, Zhang J (2020). Clinical characteristics of 138 hospitalized patients with 2019 novel coronavirus-infected pneumonia in Wuhan, China. JAMA J Am Med Assoc.

[16] García Reyes LE (2013). Acute kidney injury in patients hospitalized with COVID-19. J Chem Inf Model.

[17] Salehi S, Abedi A, Balakrishnan S, Gholamrezanezhad A (2020). Coronavirus disease 2019 (COVID-19): a systematic review of imaging findings in 919 patients. Am J Roentgenol.

[18] Pormohammad A, Ghorbani S, Khatami A et al (2021) Comparison of influenza type A and B with COVID-19: a global systematic review and meta-analysis on clinical, laboratory and radiographic findings. Rev Med Virol 31(3):e2179. 10.1002/rmv.2179 10.1002/rmv.2179 PMC7646051 33035373

[19] Ai T, Yang Z, Hou H, Zhan C, Chen C, Lv W, Tao Q, Sun Z, Xia L (2020). Correlation of chest CT and RT-PCR Testing in Coronavirus Disease 2019 (COVID-19) in China: a report of 1014 cases Tao. Radiology.

[20] Nikolai LA, Meyer CG, Kremsner PG, Velavan TP (2020). Asymptomatic SARS coronavirus 2 infection: invisible yet invincible. Int J Infect Dis.

[21] Mizumoto K, Kagaya K, Zarebski A, Chowell G, Mizumoto K, Kagaya K, Zarebski A, Chowell G (2020). Estimating the asymptomatic proportion of coronavirus disease 2019 (COVID-19) cases on board the Diamond Princess cruise ship, Yokohama. Japan Euro Surveill.

[22] Bruno R, Mondelli M, Brunetti E, Di MA, Seminari E, Maiocchi L (2020). Performance of VivaDiag COVID-19 IgM/IgG rapid test is inadequate for diagnosis of COVID-19 in acute patients referring to emergency room department. J Med Virol.

[23] National Center for Immunization and Respiratory Diseases (NCIRD) D of VD. Overview of testing for SARS-CoV-2 (COVID-19). https://www.cdc.gov/. Available from: https://www.cdc.gov/coronavirus/2019-ncov/hcp/testing-overview.html. [cited 2021 Apr 3]

[24] Chen Y, Wang AH, Yi B, Ding KQ, Wang HB, Wang JM, Shi HB, Wang SJXG (2020). Epidemiological characteristics of infection in COVID-19 close contacts in Ningbo city. Zhonghua Liu Xing Bing Xue Za Zhi.

[25] Kim SE, Jeong HS, Yu Y, Shin SU, Kim S, Oh TH, Kim UJ, Kang S-J, Jang H-C, Jung S-I, Park K-H (2020). Viral kinetics of SARS-CoV-2 in asymptomatic carriers and presymptomatic patients Seong. Int J Infect Dis J.

[26] Park HC, Kim DH, Cho A, Kim J, Yun K-S, Kim J (2021). Clinical outcomes of initially asymptomatic patients with COVID-19: a Korean nationwide cohort study. Ann Med.

[27] Petrilli CM, Jones SA, Yang J, Rajagopalan H, O’Donnell L, Chernyak Y (2020). Factors associated with hospital admission and critical illness among 5279 people with coronavirus disease 2019 in New York City: Prospective cohort study. BMJ.

[28] Gandini O, Criniti A, Ballesio L, Giglio S, Galardo G, Gianni W (2020). Serum Ferritin is an independent risk factor for Acute Respiratory Distress Syndrome in COVID-19. J Infect.

[29] Arentz M, Yim E, Klaff L, Lokhandwala S, Riedo FX, Chong M (2020). Characteristics and outcomes of 21 critically ill patients with COVID-19 in Washington State. JAMA J Am Med Assoc.

[30] Li N, Kong H, Zheng XZ, Li XY, Ma J, Zhang H (2020). Early predictive factors of progression from severe type to critical ill type in patients with Coronavirus Disease 2019: A retrospective cohort study. PLoS ONE.

[31] Ioannou GN, Locke E, Green P, Berry K, O’Hare AM, Shah JA (2020). Risk factors for hospitalization, mechanical ventilation, or death among 10 131 US veterans with SARS-CoV-2 infection. JAMA Netw open.

[32] Sokolowska M, Lukasik ZM, Agache I, Akdis CA, Akdis D, Akdis M (2020). Immunology of COVID-19: Mechanisms, clinical outcome, diagnostics, and perspectives—a report of the European Academy of Allergy and Clinical Immunology (EAACI). Allergy.

[33] Zhang J-J, Cao Y-Y, Tan G, Dong X, Wang B-C, Lin J (2021). Clinical, radiological, and laboratory characteristics and risk factors for severity and mortality of 289 hospitalized COVID-19 patients. Allergy Eur J Allergy Clin Immunol..

[34] Nasibova EM, Pashayev CN (2020) The use of Non-Invasive Ventilation (NIV) in the treatment of patients with COVID-19. J Intensive & Crit Care 6(2):5. 10.36648/2471-8505.6.2.5

[35] Carter C, Aedy H, Notter J (2020). COVID-19 disease: non- invasive ventilation and high frequency nasal oxygenation. Clin Integr Care.

[36] Covino M, Sandroni C, Santoro M, Sabia L, Simeoni B, Bocci MG (2020). Predicting intensive care unit admission and death for COVID-19 patients in the emergency department using early warning scores. Resuscitation.

